# Effects of dry period length on production, cash flows and greenhouse gas emissions of the dairy herd: A dynamic stochastic simulation model

**DOI:** 10.1371/journal.pone.0187101

**Published:** 2017-10-27

**Authors:** Akke Kok, Corina E. van Middelaar, Pim F. Mostert, Ariëtte T. M. van Knegsel, Bas Kemp, Imke J. M. de Boer, Henk Hogeveen

**Affiliations:** 1 Animal Production Systems group, Wageningen University & Research, Wageningen, the Netherlands; 2 Adaptation Physiology group, Wageningen University & Research, Wageningen, the Netherlands; 3 Business Economics group, Wageningen University & Research, Wageningen, the Netherlands; University of Illinois, UNITED STATES

## Abstract

Shortening or omitting the dry period of dairy cows improves metabolic health in early lactation and reduces management transitions for dairy cows. The success of implementation of these strategies depends on their impact on milk yield and farm profitability. Insight in these impacts is valuable for informed decision-making by farmers. The aim of this study was to investigate how shortening or omitting the dry period of dairy cows affects production and cash flows at the herd level, and greenhouse gas emissions per unit of milk, using a dynamic stochastic simulation model. The effects of dry period length on milk yield and calving interval assumed in this model were derived from actual performance of commercial dairy cows over multiple lactations. The model simulated lactations, and calving and culling events of individual cows for herds of 100 cows. Herds were simulated for 5 years with a dry period of 56 (conventional), 28 or 0 days (n = 50 herds each). Partial cash flows were computed from revenues from sold milk, calves, and culled cows, and costs from feed and rearing youngstock. Greenhouse gas emissions were computed using a life cycle approach. A dry period of 28 days reduced milk production of the herd by 3.0% in years 2 through 5, compared with a dry period of 56 days. A dry period of 0 days reduced milk production by 3.5% in years 3 through 5, after a dip in milk production of 6.9% in year 2. On average, dry periods of 28 and 0 days reduced partial cash flows by €1,249 and €1,632 per herd per year, and increased greenhouse gas emissions by 0.7% and 0.5%, respectively. Considering the potential for enhancing cow welfare, these negative impacts of shortening or omitting the dry period seem justifiable, and they might even be offset by improved health.

## Introduction

A dry period (DP) of 6 to 8 weeks is common practice in dairy cow management [1]. The DP facilitates the renewal of udder tissue and results in maximum milk yield after calving [2,3]. The DP starts with the forced cessation of milk production (drying off) and is often accompanied by ration and group changes. These procedures may cause pain (due to udder pressure), hunger, and frustration, and may therefore impair welfare of high-producing dairy cows in the period before calving [4]. Moreover, the high milk yield and limited feed intake in the first months of lactation result in a negative energy balance [5,6]. This negative energy balance is associated with metabolic disorders and reduced fertility and thus impaired animal welfare [7,8].

Shortening or omitting the DP of dairy cows can improve cow welfare through fewer management changes [4,9] and better metabolic health in early lactation [5,10]. Both shortening and omitting the DP improved the energy balance through a reduced milk yield, and a similar or increased feed intake in the subsequent lactation [5,6]. The implementation of short or no DP, however, will depend on the impact of these management strategies on factors such as herd level milk yield and farm profitability.

The effect of shortening or omitting the DP on milk yield at the herd level cannot be easily extrapolated from yield losses at the cow level. Effects of DP length also depend on herd composition, because milk yield of heifers is unaffected by DP length, whereas second parity cows experience greater reductions in milk yield than older cows [11]. Moreover, effects of DP length on milk yield are dynamic: yield reductions due to omission of the DP decreased when no DP was applied over multiple subsequent lactations [11,12]. Also, the reduction in milk yield when the DP is shortened or omitted can be compensated partly by shorter calving intervals (CI) [13], that could result from improved fertility [14,15].

The economic impact of DP length at the farm level depends on more factors than changes in total milk yield. Compared with a conventional DP, shortening and omitting the DP were found to increase milk protein content, whereas fat content appeared unaffected [16], which increases revenues when the payment system is based on milk solids. Omission of the DP improved metabolic health and reduced veterinary costs in a study on commercial dairy farms [17], although results from experimental studies on effects of DP length on disease incidence remain unclear [16]. An improvement in cow fertility could reduce economic losses [18] and involuntary culling rates [19]. Heeren et al. [20] showed that a reduction in culling rate from 37% to 24% could financially compensate an assumed reduction in milk yield of 13% due to omission of the DP.

Some studies evaluated economic impacts of shortening or omitting the DP on commercial farms [17,21], and some modelled the economic impact of varying DP lengths at the herd level using either experimental [22] or commercial data [20]. These evaluations, however, were based on comparisons of the first lactation after a change in DP length, and did not assess dynamic long-term effects on milk yield or fertility. Insight in the expected milk production at the herd level over time is valuable for informed decision-making on DP length management by farmers.

A change in DP length management might not only affect farm profitability, but also the environmental impact of milk production. One of the major global environmental challenges is climate change [23], induced by emissions of greenhouse gases (GHG). Dairy cattle are responsible for about 30% of the GHG emissions produced by the global livestock sector [24], and for about 30–40% of the emissions produced by the European livestock sector [25,26]. A major part of the GHG emissions along the milk production chain relate to the production and utilization of feed [24,25]. Shortening or omitting the DP could be accompanied by a change in ration, because a DP ration may no longer be necessary [5], and a lower daily milk yield could be matched by a reduction in energy density of the lactation ration [27]. These dietary changes can have an important influence on the level of GHGs produced [28]. Moreover, changes in milk yield and fertility might affect efficiency of milk production and, therefore, may affect GHG emissions per unit of milk produced [27,29]. Shortening or omitting the DP also improves metabolic health and could lengthen the productive life of dairy cows, which would dilute the GHG emissions related to the rearing phase [29]. To our knowledge, no evaluations of the impact of DP length on GHG emissions of milk production have been made.

The aim of this study was to investigate how shortening or omitting the DP of dairy cows affects technical and economic results at the herd level, and GHG emissions per unit of milk, using a dynamic stochastic simulation model. The effects of DP length on milk yield, CI, and cow fertility assumed in this model were based on actual performance of commercial dairy cows over multiple lactations.

## Materials and methods

### Cow simulation model

**Fig 1:**
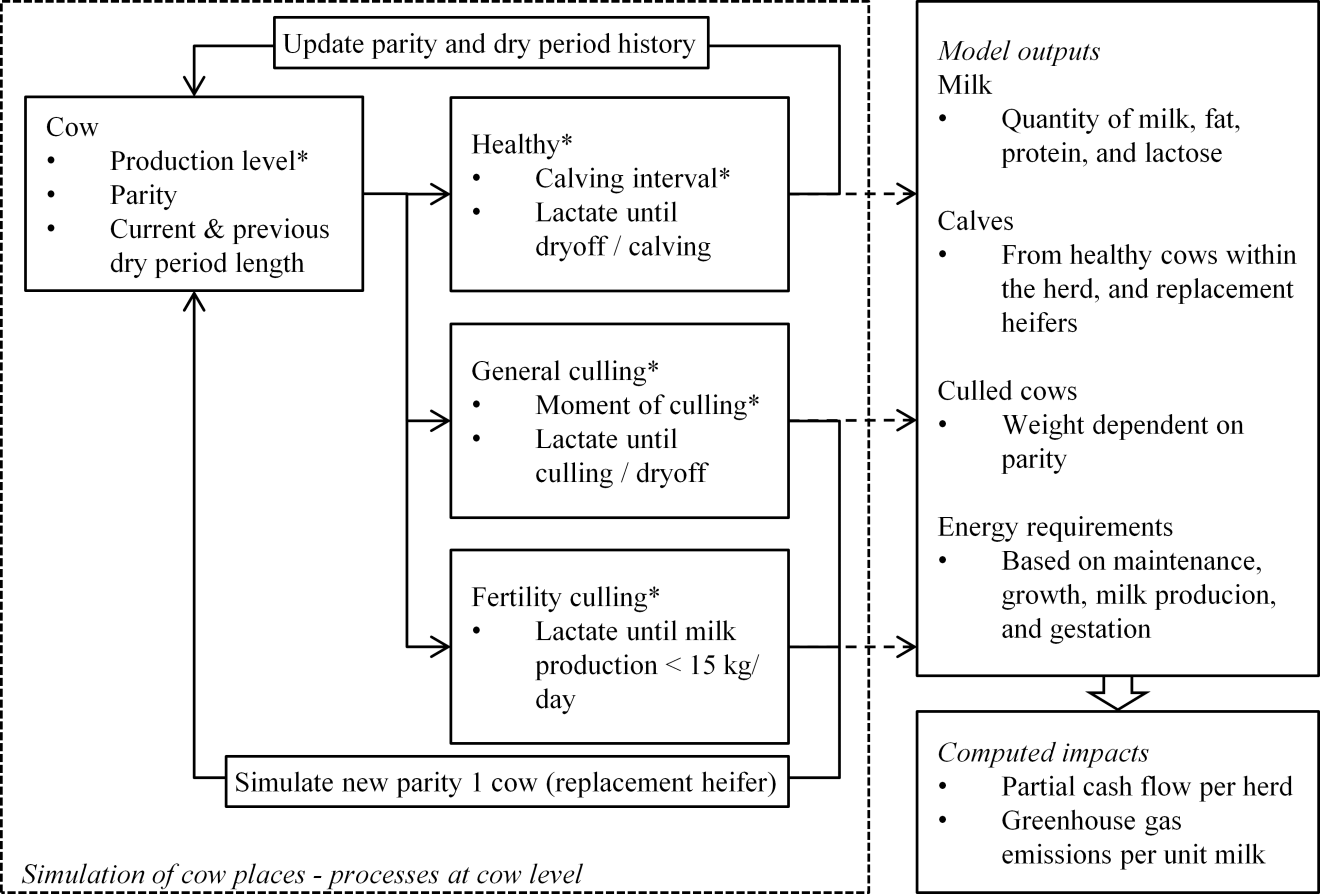
Schematic representation of the simulation model of lactations within cow places. Each cow place starts with a cow with an individual production level and parity, with a previous dry period of 56, 28, or 0 days. At the start of each lactation, cows are stochastically assigned to a healthy lactation and continuation to the next lactation, or to being culled (for general reasons or due to fertility issues) and replaced by a new heifer. Stochastic events are marked with an asterisk. Output of milk, calves and culled cows from these processes and the associated energy requirements of the cows are recorded.

A dynamic stochastic simulation model was developed in R version 3.3.1 [30] to assess how DP length affects milk production, calving, and culling at the dairy herd level over time. The model generates an average Dutch herd with 100 cow places. Each of the cow places contains one individual cow at a time, that is simulated per lactation (Fig 1). Each lactation starts with the birth of a calf, either from a healthy cow that remained in the herd or from a replacement heifer, and ends with next calving or culling of the cow. Instead of fixed daily or weekly time steps, the time steps in the developed simulation model are of a variable duration. A new time step starts when a cow calves or is culled, and when a new calendar year starts. The use of calendar years in the time steps enables the aggregation of simulated data per herd per year. When the current lactation ends before the calendar year, the whole lactation is one time step. When the current lactation exceeds the remaining number of days in the calendar year, the lactation is divided over two time steps: one until the end (365^th^ day) of this calendar year, and another that starts in the next year and ends at calving or culling. Per time step per cow place, the model records the produced milk, calves, and culled cows, and computes the associated energy requirements.

To simulate lactations of cows in cow places over time, lactation curves, CI, and culling (probability and timing) were modelled. Input values for each DP length were derived from milk production data (2007–2015) from 16 Dutch dairy farms that deliberately shorten or omit the DP since 2010/2011 and applied conventional DP (≥ 6 weeks) before [11,13]. The modelling and input values for milk production, CI and culling are described in more detail below.

#### Milk production

Lactation curves were used to simulate milk production of cows after a DP of 56, 28, or 0 days. Individual milk production (MP) in kg of cow *i* in parity *j* with DP category *l* at each day in milk (DIM) was calculated as:

$$MP_{ijl}=a_{jl}+b_{j}\times DIM+c\times exp\left(\text{-}k\times DIM\right)+RPL_{i}\times ADY_{jl,}$$

where RPL_i_ is the relative production level of cow *i*; ADY_jl_ is the average daily 305-d yield in kg milk of a cow in parity *j* with DP category *l*, and a, b, c, and k model the shape of the lactation curve [31].

The RPL was drawn from a normal distribution with a mean of 0 and standard deviation of 0.1, to reflect natural variation in milk production from about 80% to 120% of the average lactation [18]. All other parameters in the lactation curve were fixed (Table 1).

**Table 1:** Model inputs for individual lactation curves per dry period length.

Parity	DP length (days)	ADY (kg)	a	b	Fat (%)	Protein (%)	Lactose (%)
1	-	23.9	31.6	-0.0447	4.48	3.55	4.62
2	56	28.9	40.6	-0.0708	4.50	3.59	4.53
	28	25.9	37.6	-0.0708	4.64	3.75	4.55
	0	22.1	33.8	-0.0708	4.81	3.93	4.51
>2	56	30.5	44.1	-0.0835	4.51	3.51	4.48
	28	27.7	41.3	-0.0835	4.49	3.62	4.48
	56–0^a^	24.4	38.0	-0.0835	4.60	3.71	4.41
	0–0^a^	27.0	40.6	-0.0835	4.53	3.62	4.41

The average daily 305-d milk yield (ADY); parameters a and b of the Wilmink lactation curves; and fat, protein, and lactose content of the milk per parity class per dry period (DP) category. Parameter c was -16.1 and parameter k was 0.06.

^a^56-0: no DP in the current lactation after a DP of 56 days in the previous lactation; 0–0: no DP in the current lactation after no DP in the previous lactation.

Production level (parameter a) was assumed to be affected by parity class and DP category [6,11]; persistency (parameter b) was assumed to be affected by parity only [12]; parameter c was assumed not to be affected by parity or DP length (best model fit based on BIC values); and parameter k was set to 0.06 [11]. To compute values for parameters a, b, and c, Wilmink lactation curves [31] were fitted on the raw test-day milk records per parity class per DP category, using a mixed model in SAS version 9.3 [11,13,32] (S1 Table). The model included random effects on a, b, and c for repeated measures per cow lactation assuming unstructured covariance [11]. Milk records were grouped in the parity classes 1, 2, and >2 to model the difference in persistency and effect of DP length on parity, and in DP categories standard DP (6–12 weeks), short DP (3–5 weeks), and no DP (0–2 weeks), to represent the model DP lengths of 56, 28, and 0 days. Because the effect of no DP on milk production depends on the previous DP length [11], the last category was split up in two subcategories: no DP preceded by a standard DP, and no DP for multiple lactations.

Average protein, fat, and lactose content of the produced milk were calculated per parity class per DP category, and used to parameterise the milk composition of the simulated lactation curves. Previous research already indicated interaction effects of parity and DP length for these variables [6]. Milk yield of each cow was computed per cow space per time step, using the integral of the MP function. If the individual daily milk production reached 0 kg before the designated moment of dry-off, occurrence of the spontaneous dry-off was recorded.

#### Calving interval

The model randomly assigned a CI to each lactation based on parity class and DP category, except when the cow was culled due to fertility issues. It was assumed that DP length affected CI, because a reduction in CI due to shortening or omitting the DP has been reported on commercial farms [13,17,21]. The CI data in this model were taken directly from the same dataset as the milk production data, clustered per parity class per DP category (Table 2) [11,13]. Calving intervals exceeding 518 days were discarded, to reflect that attempts of insemination would cease 34 weeks after calving [33] to reduce economic losses due to longer CI [18].

**Table 2:** Model inputs for calving intervals and fertility culling per dry period length.

Parity	DP length (days)	Median CI	P5	P95	n	P_fertility culling_
1	-	374	327	477	2,348	0.080
2	56	381	330	487	1,116	0.075
	28	365	325	482	495	0.052
	0	359	316	464	342	0.039
>2	56	385	333	489	1,850	0.078
	28	378	328	480	629	0.074
	0	370	321	473	573	0.037

Distribution of calving interval (CI) records (median days, 5 and 95 percentiles, n) used as model input, and fraction of records exceeding 518 days (P_fertility culling_), per parity class per dry period (DP) category. Records exceeding 518 days were excluded from the dataset before descriptives were computed.

#### Culling

Within a cow space, each lactation of a cow is stochastically assigned to one of three categories: healthy, culled due to fertility issues (fertility culling), or culled for other reasons (general culling). When a cow is culled, she is replaced by a heifer that is assumed to calve and to enter the herd the following day. This is a simplified version of the assumption that some heifers enter the herd before a cow is culled (overstocking), whereas others replace culled cows with a possible delay, thus leaving a cow space empty for some time [33].

The probability of fertility culling varied based on parity class and DP category (Table 2). It was assumed that CI in the unfiltered dataset that exceeded 518 days would result in fertility culling in the model [33]. Therefore, the probability of fertility culling per parity class per DP category was set equal to the percentage of CI exceeding 518 days in the unfiltered dataset. This was about 8% of the lactations for cows with a standard DP. Cows assigned to fertility culling did not become pregnant and were culled when their milk production dropped below 15 kg per day [33].

The probability of general culling was constant across parities and DP lengths, and was set at 0.22 per lactation to create an overall culling rate (fertility culling and general culling) of about 30% for cows with a standard DP [19]. General culling occurred at a certain fraction of completion of a cow’s assigned CI, drawn from a distribution with a positive skew and a median fraction of 0.17 (beta distribution with parameters a = 1.3, b = 5 [33]).

### Simulation and model outputs

The model herd started on day 1 with 100 cows, with a fixed number of cows from parity 1, 2, 3, 4, and >4 (30, 21, 15, 10, and 24, respectively) to reflect a 30% culling rate. The model was run for 5 years with a standard DP of 56 days to introduce variation in initial herds. For each DP length (56, 28, and 0 days), 50 herds were simulated, to get insight in the degree of variation in technical performance due to stochasticity. At the start of the 6^th^ year, average herd composition of the 150 herds was equal to the input herd composition (29.8, 20.7, 14.9, 10.4, and 24.2 cows in parity 1, 2, 3, 4, and >4, respectively), with SD of 3.0 to 5.1 cows per parity class. The 6^th^ year was used as a baseline situation (year 0), and scenarios with a DP of 28 or 0 days were implemented from the start of the 7^th^ year (year 1 after change in DP length). Each herd was simulated for 5 years following implementation of the new DP length in year 7. Preliminary data showed that additional herds hardly changed the average and range of model results.

#### Sensitivity analysis

A sensitivity analysis was performed to assess the effect of general culling rate and of assumed effects of DP length (on milk production, CI, and fertility culling) on model results. The probability of general culling was 0.22 in the model, resembling an overall culling rate of about 30% for cows with a standard DP. Culling rates for Dutch dairy farms, however, commonly vary from 20% to 35% between farms [19]. A change in culling rate could affect the effect of DP length on milk yield through a different herd composition, and through more lactations being terminated in early lactation. To assess this impact, the probability of general culling was changed to 0.12, 0.17, and 0.27 in the sensitivity analysis, creating overall culling rates of 20%, 25%, and 35% for cows with a standard DP, respectively.

Dry periods of 28 and 0 days were assumed to reduce milk production, shorten CI, and reduce fertility culling compared with a DP of 56 days. Reductions in milk production varied between 2.8 to 6.8 kg milk per day in the model input, depending on parity and DP length. In the sensitivity analysis, the impact of a greater or lesser reduction in milk production was assessed. To assess the impact of shorter CI and reduced fertility culling in case of a DP of 28 or 0 days, two more scenarios were assessed in which CI or fertility culling was not affected by DP length (i.e. input values from the DP of 56 days were used).

#### Energy requirements and ration composition

Energy requirements for maintenance, milk production, growth (for parity 1 and 2), and gestation were computed per time step according to the Dutch net energy evaluation system in VEM (1,000 VEM = 6.9 MJ of net energy) [34], using the parity, weight, milk production, and pregnancy status of the cow [35]. Maintenance requirements are 42.4 VEM per kg^0.75^ of body weight [35]. Body weight linearly increased from 540 kg at first calving to 595 kg at second calving, and to 650 kg at third calving. Cows had fixed energy requirements for growth in parity 1 (660 VEM per day) and parity 2 (330 VEM per day) and in the last 4 months of pregnancy (450, 850, 1,500, and 2,700 VEM per day, respectively) [35]. It was assumed that the lactating cows were grazing for 8 hours per day in the summer period for 170 days per 365 days [36], and that grazing increased energy requirements for maintenance by 6.7% [35]. It was assumed that dry cows were housed indoors, which is, based on the experience of the authors, generally the case.

Feed requirements were computed using an average Dutch ration for (lactating and dry) dairy cows in the summer and winter period (Table 3) [36]. Roughage consisted of grass, grass silage and maize silage, and was supplemented with byproducts and concentrate [36,37]. In case of a DP of 28 or 0 days, a second ration was composed, in which the energy content of the average Dutch ration was reduced to simulate a potential change in feeding management [27,38]. This was done by first computing the ration for an average day for a cow with a DP of 56 days, based on her average energy requirements per day [35,39]. Subsequently, the amount of concentrate was reduced to match the average energy requirements per day of cows in herds with DP of 28 or 0 days. To keep a comparable intestinal digestible protein to net energy ratio in the ration, standard concentrate was exchanged for protein-rich concentrate [40]. Effects of a DP of 28 or 0 days are presented for the average Dutch ration; the impact of the potential reduction in concentrate is presented separately. Because the average daily energy requirements were very similar for herds with a DP of 28 or 0 days from year 3 after the change in DP length onwards, the alternative ration was computed using the average energy requirement of herds with a DP of 28 or 0 days from year 3 to year 5, based on a reduction of 2.2 MJ per cow per day in winter and 1.8 MJ in summer compared with herds with a DP of 56 days.

**Table 3:** Ration specifications of the average Dutch ration and the reduced concentrate ration in the model.

	Average Dutch ration^a^		Reduced concentrate ration	
	Winter	Summer	Winter	Summer
Composition (% of DM)				
• Grass	0.0	39.0	0.0	39.5
• Grass silage	55.1	25.2	55.9	25.6
• Maize silage	13.7	10.9	13.9	11.0
• Wet by-products^b^	4.8	3.8	4.9	3.8
• Normal concentrate^c^	19.7	21.1	17.4	19.6
• Protein concentrate^c^	6.8	0.0	7.9	0.5
Net energy (MJ/ kg DM)^d^	6.5	6.8	6.5	6.8
GHG emissions (kg CO_2_e per t DM)^e^				
• Feed production	468	470	463	466
• Enteric fermentation	574	585	572	584

Composition and specifications of the average Dutch ration for dairy cows and of a ration reduced in concentrate designed for herds with a DP of 28 or 0 days, split in a winter ration (195 days per year) and a summer ration (170 days per year).

^a^Based on [36]

^b^Wet by-products include brewers grain, potato peel, potato pulp, and maize gluten meal

^c^Protein concentrate has more soybean hulls, palm kernel expeller, and distillers grains and solubles than standard concentrate per kg DM, and less maize and wheat middlings.

^d^Calculated with the Dutch net energy evaluation (VEM) system [34]

^e^Based on [39]

### Calculation of partial cash flows

A partial cash flow analysis was performed to assess economic consequences of shortening or omitting the DP at the herd level. This analysis included revenues from sold milk, calves, and culled cows, and costs from buying or producing feed and rearing youngstock (Table 4).

**Table 4:** Costs and revenues of parameters used to compute partial cash flows.

	Value (€)
Milk revenues (per 100 kg solids)^a^	
• Protein	576.48
• Fat	288.25
• Lactose	57.65
Calves revenues (per animal)^b^	
• Female calf	51.00
• Male calf	109.00
Culled cows (per kg meat)^b^^,^^c^	2.32
Replacement heifer (per animal)^b^	969.00
Feed costs (per t DM)^d^	
• Summer ration	167.80
• Winter ration	202.30
• Summer ration low concentrate	167.00
• Winter ration low concentrate	202.00

^a^This results in €35.32 per 100 kg milk with average solids content (3.47% protein, 4.41% fat and 4.51% lactose), corresponding to the average Dutch milk price 2008–2016 [41]

^b^Average of Dutch values from 2008–2016 [42]

^c^Assumed dressing percentage of 60% [33]

^d^Based on [43]

Milk revenues were according to the Dutch payment system based on milk solids (value of protein:fat:lactose of 10:5:1), using the average Dutch milk price over the period 2008–2016 [41]. Revenues for surplus calves and culled cows, as well as the costs of raising a heifer were computed from yearly values over the period 2008–2016 taken from Wageningen Economic Research [42]. It was assumed that 50% of the calves were male and 50% of the calves were female; and that the number of female calves retained for replacement equalled 113.4% of the number of culled cows, to account for 13.4% mortality during the rearing phase; and that 7% of surplus calves died on farm [43]. Feed costs were calculated from Dutch feed prices per feedstuff [43]. Partial cash flows were computed per herd per year, and expressed as difference in partial cash flow compared with a DP of 56 days.

### Calculation of greenhouse gas emissions

To assess the impact of shortening or omitting the DP on GHG emissions, a life cycle approach was used. Emissions of carbon dioxide (CO_2_), methane (CH_4_), and nitrous oxide (N_2_O) were computed for all processes along the milk production chain that were assumed to be affected by a change in DP length, including feed production, enteric fermentation, and manure management. Accounting for feed production [39], enteric fermentation [39], manure management [44–48] and mortality in the rearing phase (assuming an age at first calving of 24 months) [43], GHG emissions related to the rearing of young stock were estimated to be 4,905 kg CO_2_ equivalents per replacement heifer. GHG emissions of the dairy cows were computed from the model results using the same method. Emissions related to feed production included: production of inputs (e.g. fertilizer and machinery), cultivation, harvest, and processing of the feed products, and transport to farms [39]. Economic allocation was used in case of a multiple output process (e.g. production of soybean meal also results in soybean oil), because feed ingredients and their co-products can be used in many pathways (e.g. feed, food, biofuel) and have distinct characteristics (nutritional values) which makes system expansion and physical allocation undesirable [49,50]. Emissions related to enteric fermentation were calculated with feed specific emission factors [39].

Emissions related to manure management were calculated from the volume of manure and the nitrogen excretion. Nitrogen excretion was computed as the difference between nitrogen intake from feed and nitrogen retention for milk production, growth, and gestation [48]. Moreover, it was assumed that during the grazing period, 1/3 of the manure was excreted during grazing (8 hours per day), and 2/3 was excreted in stables and subsequently stored; which resulted in different GHG emission factors (S2 Table). Factors for N_2_O, NH_3_, NO_x_ and CH_4_ emissions and NO_3_^-^ leaching from manure on pasture and in the stable were taken from Dutch national inventory reports [44–47], and emission factors from NH_3_, NO_x_ and NO_3_^-^ to N_2_O (i.e. indirect N_2_O emissions) were taken from IPCC [51]. All GHG emissions were converted to CO_2_ equivalents, based on their equivalence factor in terms of CO_2_ (100-year time horizon): 1 for CO_2_, 28 for biogenic CH_4_, 30 for fossil CH_4_, and 265 for N_2_O [52]. Total GHG emissions were expressed as CO_2_ equivalents per kg fat-and-protein-corrected milk (FPCM). System expansion was used to account for the production of meat from calves and cows [29]. The production of meat from surplus calves (as white veal) and cows was assumed to substitute the production of other meat on the basis of kg edible product. The model accounted for additional GHG emissions related to rearing (calves), transport and slaughter [29], and for avoided GHG emissions related to the production of poultry, pigs and cows elsewhere [53].

## Results

### Technical results

**Fig 2:**
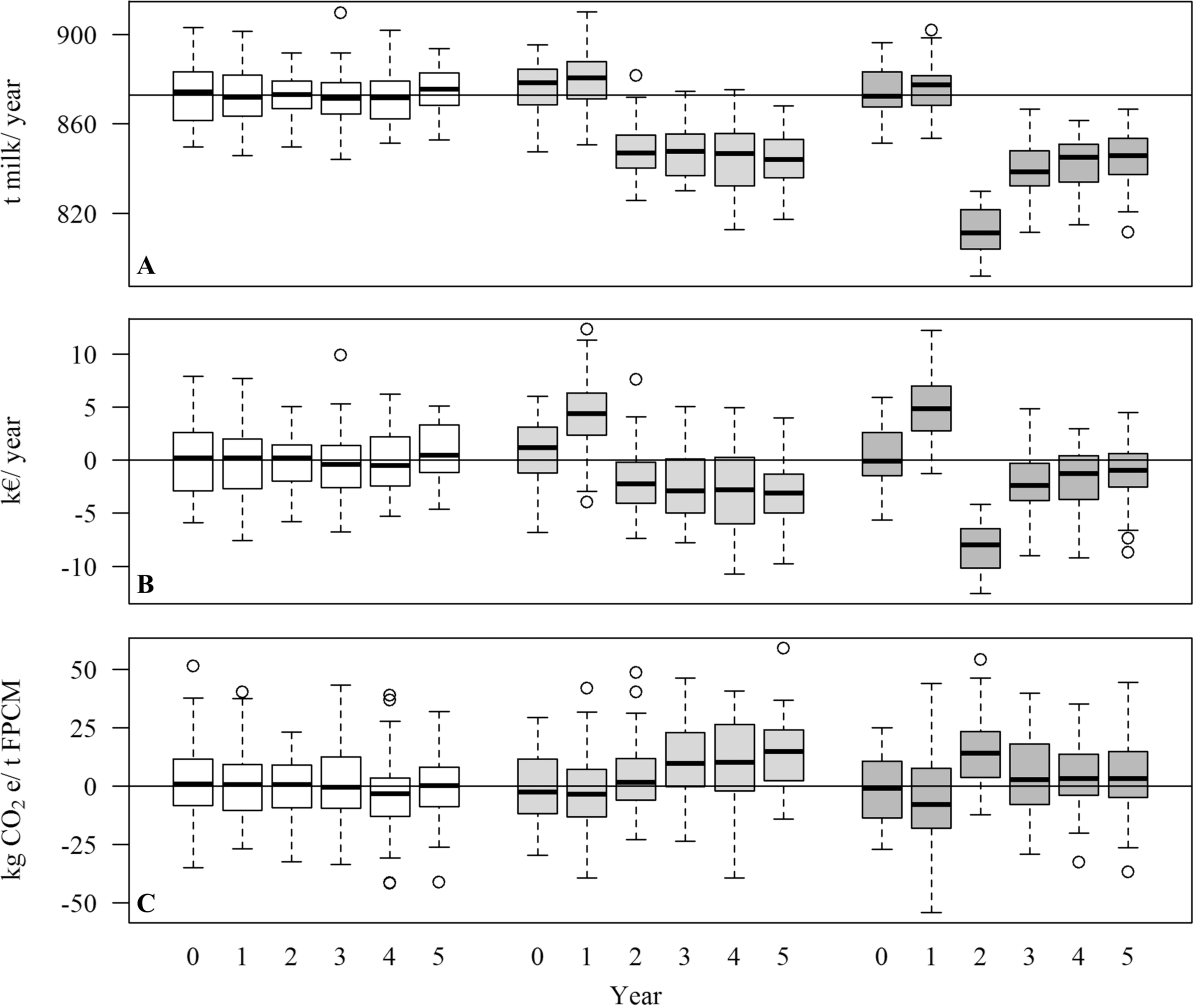
Impact of dry period length on milk production, partial cash flow, and greenhouse gas emissions. (A) Milk production per herd per year, (B) difference in partial cash flow, and (C) difference in greenhouse gas emissions per t fat-and-protein-corrected milk (FPCM) compared with mean of herds with a dry period of 56 days (reference line), over a period of 6 years for herds with a dry period of 56 days (white box plots), and herds that switched to a dry period of 28 days (light grey) or 0 days (dark grey) in year 1, following a dry period of 56 days in year 0.

The milk production, number of calves born and cows culled per herd (with 100 cows) per year are presented in Table 5. In the baseline year, all herds applied a DP of 56 days, and the average milk production per herd varied 0.4% between the DP strategies due to stochasticity (from 873,285 kg to 876,433 kg; n = 50 herds each). Herds that switched to a DP of 28 days had a higher average milk production in the first year the strategy was applied (+7,283 kg; +0.8%), and then seemed to stabilize at an average milk production of 845,987 kg per year from year 2 until year 5, which was 3.1% lower than herds with DP of 56 days (-37,869 kg per year). Herds that switched to a DP of 0 days also had a slightly higher average milk production than herds with a DP of 56 days in the first year the strategy was applied (+4,244 kg; +0.5%). In year 2, the average milk production was 812,275 kg, which was 6.9% lower than of herds with DP of 56 days (-60,117 kg). From year 3 until year 5, average milk production of herds with a DP of 0 days was 842,360 kg, which was 3.5% lower than herds with a DP of 56 days (-30,452 kg per year). Variation between herds was similar for different DP lengths (Fig 2A), with an average coefficient of variation of 1.4% on herd averages per year.

**Table 5:** Technical simulation results: Average production, days dry and energy requirements per herd per year.

	DP (days)	Year 0		Year 1		Year 2		Year 3		Year 4		Year 5	
Output variable		Avg	SD	Avg	SD	Avg	SD	Avg	SD	Avg	SD	Avg	SD
Milk (t)	56	873	13	872	12	872	10	871	11	872	12	875	10
	28	876	11	880	13	848	12	848	12	844	15	845	11
	0	875	10	877	11	812	10	839	13	843	12	845	12
FPCM^a^ (t)	56	936	14	935	13	935	11	934	12	934	12	937	11
	28	939	12	946	14	914	12	914	13	910	16	911	11
	0	937	11	947	12	885	10	911	13	915	12	917	13
calves (n)	56	114	7	114	5	113	5	114	6	112	6	114	4
	28	114	6	114	6	115	6	117	6	116	6	117	5
	0	113	5	114	5	118	5	118	6	118	6	118	7
Cows culled (n)	56	34	7	34	6	33	5	34	7	32	6	34	5
	28	34	6	35	6	32	6	35	6	34	6	35	5
	0	33	5	35	7	32	6	32	6	32	5	32	6
Days dry^b^ (n)	56	45	2	45	2	45	2	45	2	45	2	44	2
	28	44	2	22	1	23	1	23	1	23	1	23	1
	0	45	2	4	1	1	1	1	1	1	1	1	1
NE winter^c^ (MJ)	56	122	1	121	1	121	1	121	1	121	1	122	1
	28	122	1	122	1	119	1	119	1	119	1	119	1
	0	122	1	122	1	117	1	119	1	119	1	120	1
NE summer^c^ (MJ)	56	126	1	126	1	126	1	126	1	126	1	126	1
	28	126	1	127	1	124	1	124	1	124	1	124	1
	0	126	1	127	1	122	1	124	1	124	1	125	1

Technical simulation results for herds with a dry period (DP) of 56 days in year 0, and a DP of 56, 28, or 0 days from year 1. Average values per herd (100 cows) per year and SD are presented (n = 50 herds per DP length).

^a^FPCM = fat-and-protein-corrected milk

^b^Total days without milk production per cow per year

^c^NE = Net energy requirement per cow per day

On average, 114 calves were born per herd per year in case of a DP of 56 days. From year 2, the number of calves born increased by 3 calves per year when a DP of 28 days was applied, and by 5 calves when a DP of 0 days was applied, compared with a DP of 56 days. Variation in the number of calves born between herds was similar for different DP lengths, with an average coefficient of variation of 5.0%. On average, 34 cows were culled per herd per year in case of a DP of 56 days. The number of culled cows appeared to be about 1 less when a DP of 0 days was applied, but variation between herds was large with an average coefficient of variation of 17.7%. In case of a target DP length of 0 days, some cows spontaneously dried themselves off, resulting in an average of about 1 day dry per cow per year.

**Fig 3:**
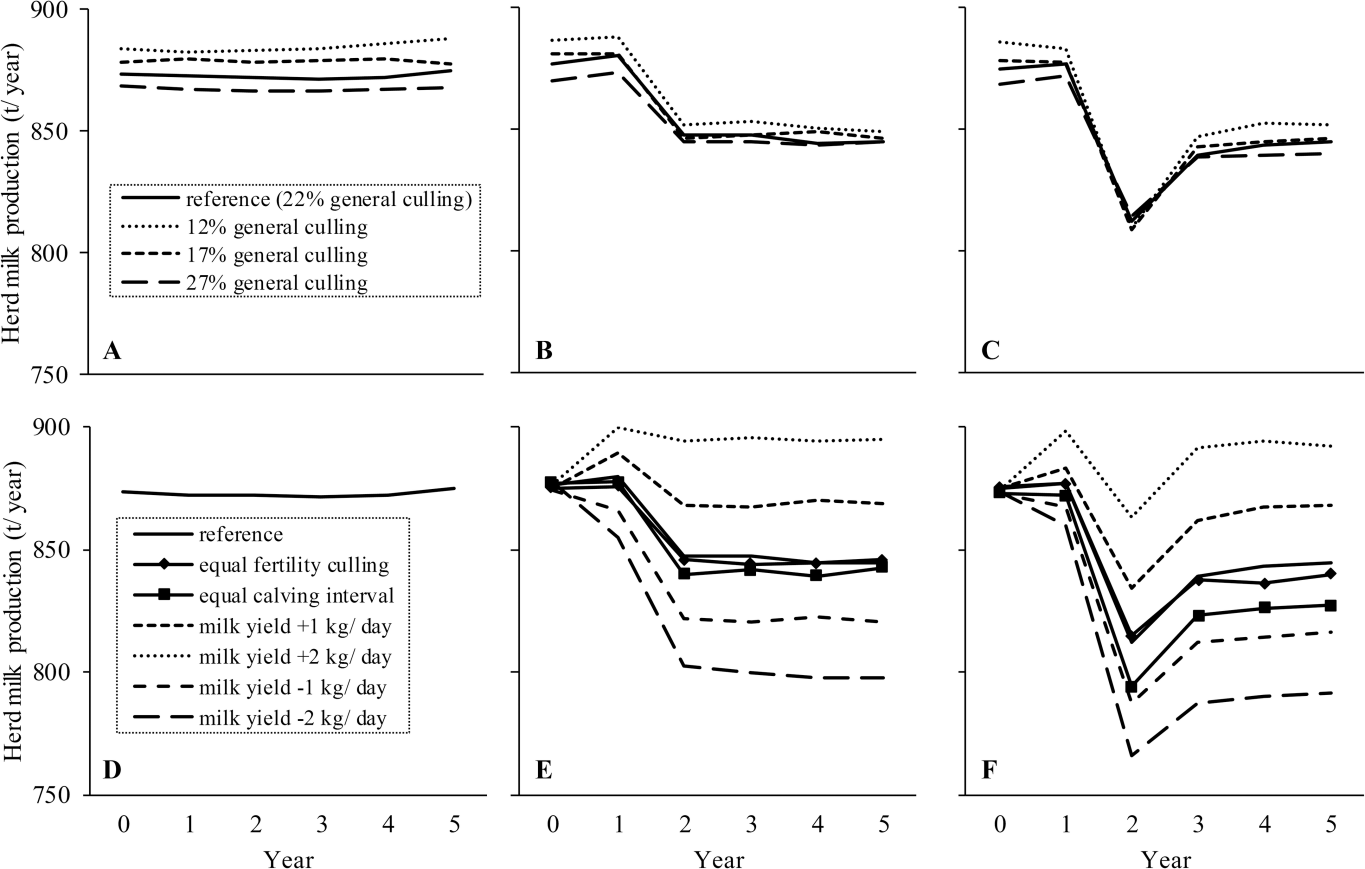
Impact of model assumptions regarding culling and effects of dry period length on milk production. Average milk production per herd per year for different general culling rates with a dry period of 56 (A), 28 (B), or 0 days (C); and for 1 and 2 kg per day lesser or greater milk reductions, no effect on fertility culling, or no effect on CI compared with a dry period of 56 days (D) in case of a dry period of 28 (E) or 0 days (F). Results are shown for the year before and 5 years following a switch to a dry period of 28 or 0 days in year 1.

Effects of the model assumptions for general culling rate and for effects of DP length (on milk production, CI, and fertility culling) on average herd milk production are presented in Fig 3. For all DP lengths, a lower general culling rate resulted in a higher herd milk production (Fig 3A–3C), but the impact of a change in general culling rate was smaller in case of a DP of 28 or 0 days. A reduction in general culling rate could not compensate milk losses due to a DP of 28 or 0 days. Assuming different milk reductions due to a DP of 28 or 0 days had a large impact on herd milk production, and lessening milk reductions by 2 kg per day in lactation resulted in higher herd milk production with a DP of 28 or 0 days than with a DP of 56 days (Fig 3D–3F). Assuming no reduction in fertility culling compared with a DP of 56 days hardly reduced herd milk production for a DP of 28 or 0 days. Assuming no shortening of CI slightly reduced herd milk production in case of a DP of 28 days, and considerably reduced herd milk production in case of a DP of 0 days.

### Economic impact: Partial cash flow

In the reference scenario, where DP length affected milk production, CI, and fertility culling, a DP of 28 or 0 days increased the average partial cash flow in the first year the strategy was applied (Fig 2B). From the second year onwards, however, both strategies resulted in a decreased cash flow compared with a DP of 56 days. In case of a DP of 28 days, losses from year 2 to year 5 averaged €2,608 per herd per year. In case of a DP of 0 days, losses were most severe in year 2 at €8,138 per herd, after which losses from year 3 to 5 averaged €1,705 per herd per year.

Results of the sensitivity analysis are expressed as change in cash flow in euros per year compared with herds with a DP of 56 days and a general culling rate of 22% (Table 6). For all DP lengths, a lower culling rate resulted in a higher partial cash flow. However, the difference in partial cash flow between 12% and 27% general culling was smaller for a DP of 28 or 0 days than for a DP of 56 days. Regarding the assumed effects of a DP of 28 or 0 days, partial cash flows were least sensitive to changes in the probability of fertility culling, quite sensitive to changes in CI, and most sensitive to changes in milk reduction. A reduction in concentrate in the ration in case of a DP of 28 or 0 days decreased feed costs by €132 per year at the herd level.

**Table 6:** Economic results: Impacts of dry period length and model assumptions on partial cash flows.

	Year 1			Year 2			Year 3		
Parameter settings^a^	56	28	0	56	28	0	56	28	0
12% general culling	3,592	7,359	7,892	3,887	220	-7,757	4,313	1,083	1,050
17% general culling	2,405	5,142	5,769	2,085	-1,672	-8,450	2,578	-1,186	-576
22% general culling	REF	4,187	5,091	REF	-1,900	-8,138	REF	-1,827	-1,926
27% general culling	-2,074	1,807	2,760	-2,373	-3,733	-8,620	-2,050	-3,316	-2,936
Equal fertility culling		3,122	5,099		-2,547	-7,877		-2,829	-2,837
Equal calving interval		3,498	3,900		-4,340	-12,983		-3,390	-6,262
Milk yield +1 kg/ day		6,682	7,018		3,339	-1,960		3,598	4,330
Milk yield +2 kg/ day		9,617	10,975		10,142	5,503		11,047	12,192
Milk yield -1 kg/ day		342	2,284		-9,126	-15,178		-9,066	-9,040
Milk yield -2 kg/ day		-2,376	215		-14,174	-21,309		-14,625	-16,010

Average difference in partial cash flow in euros per herd (100 cows) per year compared with a dry period of 56 days and 22% general culling for different parameter settings, following a change in dry period length to 28 or 0 days in year 1. Partial cash flows were computed as milk, meat, and calf revenues minus feed costs and youngstock costs.

^a^Parameter settings were changed from the reference (REF) of 22% general culling to different general culling rates for all dry period lengths; and from the assumed reduction in fertility culling, shortening of calving interval, and quantity of milk reduction in case of a dry period of 28 or 0 days to: no effect of dry period length on fertility culling, no effect of dry period length on calving interval, or a 1 or 2 kg per day lesser or greater reduction in milk yield (assuming the same ration for all dry period lengths).

### Environmental impact: Greenhouse gas emissions

In the reference scenario with a DP of 56 days, GHG emissions of milk production were on average 943 kg CO_2_ equivalents per t fat-and-protein-corrected milk (FPCM). On average over the 5 years, GHG emissions increased by 8 kg CO_2_ equivalents per t FPCM in case of a DP of 28 days, and by 5 kg CO_2_ equivalents per t FPCM in case of a DP of 0 days. These average increases were minor compared with the between-farm variation within DP strategies (Fig 2C). From year 3 onwards, GHG emissions per t FPCM were lower for a DP of 0 days than for a DP of 28 days.

The sensitivity analysis showed that a lower culling rate resulted in lower GHG emissions per t FPCM for all DP lengths (Table 7). The effect of culling on GHG emissions was larger than any of the assumed effects of changes in DP length. Considering the assumed effects of a DP of 28 or 0 days, emissions seemed hardly sensitive to the change in CI, in case of a DP of 0 days quite sensitive to the probability of fertility culling, and most sensitive to changes in milk yield. A reduction in concentrate in the ration in case of a DP of 28 or 0 days reduced GHG emissions of milk production by 4 kg CO_2_ equivalents per t FPCM.

**Table 7:** Environmental results: Impacts of dry period length and model assumptions on greenhouse gas emissions.

	Year 1			Year 2			Year 3		
Parameter settings^a^	56	28	0	56	28	0	56	28	0
12% general culling	-42	-42	-45	-39	-29	-19	-43	-35	-41
17% general culling	-22	-25	-26	-19	-14	-3	-25	-16	-19
22% general culling	REF	-3	-5	REF	5	17	REF	9	3
27% general culling	23	16	21	30	33	36	25	29	24
Equal fertility culling		0	-7		10	26		11	13
Equal calving interval		0	-4		13	22		10	4
Milk yield +1 kg/ day		-5	-9		0	2		-3	-11
Milk yield +2 kg/ day		-13	-12		-6	-6		-14	-19
Milk yield -1 kg/ day		6	1		24	29		21	10
Milk yield -2 kg/ day		7	4		31	44		30	30

Average change in greenhouse gas emissions in kg CO_2_ equivalents per t fat-and-protein-corrected milk per herd (100 cows) per year compared with a dry period of 56 days and 22% general culling for different parameter settings, following a change in dry period length to 28 or 0 days in year 1.

^a^Parameter settings were changed from the reference (REF) of 22% general culling to different general culling rates for all dry period lengths; and from the assumed reduction in fertility culling, shortening of calving interval, and quantity of milk reduction in case of a dry period of 28 or 0 days to: no effect of dry period length on fertility culling, no effect of dry period length on calving interval, or a 1 or 2 kg per day lesser or greater reduction in milk yield (assuming the same ration for all dry period lengths).

## Discussion

The aim of this study was to investigate how shortening or omitting the DP of dairy cows affects technical and economic results at the herd level, and GHG emissions per unit of milk, using a dynamic stochastic simulation model. Considering the technical results, a change in DP length had a clear impact on milk yield, whereas the impact on number of calves born and cows culled was smaller than the variation between herds with the same DP. In the first year of application of a DP of 28 or 0 days, milk yield of the herd increased compared with the conventional DP of 56 days. This can be explained by the fact that all cows in the herd started the year in a lactation after a conventional DP, and this lactation was prolonged because of the shortened or omitted DP. The resulting additional yield was greater than the milk losses of cows that already entered their next lactation in year 1. Milk yields of herds with a DP of 28 days decreased by 3.1% from year 2 of the strategy, compared with a DP of 56 days. At this point, most multiparous cows started lactations following a DP of 28 days, and faced associated reductions in milk production. Milk yield of herds with no DP (0 days) decreased by 6.9% in year 2 and by on average 3.5% per year from year 3 onwards, compared with a DP of 56 days. The higher milk yield from year 3 onwards can be explained by the milk yield input: cows in their second or later lactation after omission of the DP had a higher milk yield than cows in the first lactation after omission of the DP [11,12]. From the third year onwards, most older cows will have lactations preceded by two omitted DP.

The decrease in milk production at the herd level in the current study (3.1% for a short and 3.5% for no DP) is much smaller than the reported milk losses in individual lactations following a shortened or omitted DP (4.5% for a short and 19% for no DP) [16], and smaller than calculated milk losses based on individual lactations after correcting for additional milk yield before calving and improved fertility (3.1% in parity 2 and 4.0% in parity >2 for a short DP and 11% in parity 2 and 8.0% in parity >2 for no DP) [11]. Two factors that contribute to these lesser reductions in milk yield at the herd level are the presence of first parity cows and incomplete lactations due to culling. The lactation of a cow in first parity starts when the first calf is born, and therefore is not affected by a change in DP length. This means that roughly a third of the herd does not face reductions in milk production due to a short or no DP. Culling implies that lactations are terminated earlier in lactation. Before culling, cows with a DP of 28 or 0 days have realised a considerable additional milk production in the 8 weeks before calving, whereas cows with a DP of 56 days have been dry. This outweighs a lower milk production from calving until culling and results in a higher effective lactation yield (daily milk yield from 60 days before calving until the moment of culling) for cows with a shorter DP (Table 8; [13]). As a consequence, general culling had a larger impact on milk production of herds with a DP of 56 days than of herds with a DP of 28 or 0 days, which lessened reductions in milk yield compared with a DP of 56 days.

**Table 8:** Effective lactation yields of healthy and culled cows per dry period length.

		Healthy		Fertility culling		General culling	
Parity	DP category	ELY	CI	ELY yield	Cull day	ELY	Cull day
1	-	23.5	323	19.4	372	12.4	77
2	56	24.4	331	23.7	359	18.6	81
	28	22.7	319	22.9	323	19.1	75
	0	21.0	309	21.7	267	21.0	73
>2	56	25.4	335	25.2	350	19.9	80
	28	23.7	326	24.0	317	20.0	78
	56–0^a^	22.1	322	23.2	276	21.8	78
	0–0^a^	23.9	319	24.2	308	21.3	77

Average effective lactation yields in kg per day (ELY), calving intervals (CI) and day of culling for lactations of cows that calved again (healthy), cows that were culled for fertility issues (fertility culling), and cows that were culled for other reasons (general culling). Effective lactation yield was computed as kg fat-and-protein corrected milk per day from 60 days before calving until 60 days before next calving or until culling.

^a^56-0: no DP in the current lactation after a DP of 56 days in the previous lactation; 0–0: no DP in the current lactation after no DP in the previous lactation.

The change in milk production over time after switching to no DP can be important knowledge for decision-making by dairy farmers, and illustrates the relevance of using a dynamic model. It is known from practice that some farmers have quit omitting the DP within 2 years because of a too low milk production [54], whereas they might have continued–or never started–the strategy if they had been prepared for these dynamics.

The model also provides insight in days dry per cow per year, accounting for herd composition, CI, and culling. With the data used in this study, cows with a short and no DP lactated 22 and 44 days per year more than cows with a standard DP, respectively. From this, effects of overall yield level on performance can be extrapolated: a 1 kg lower daily milk yield would result in 364 kg less milk per cow per year in case of no DP, and in 320 kg less milk per cow per year in case of a standard DP. In this way, the overall milk reductions of omitting the DP compared with a standard DP will be 44 kg per cow per year greater if production levels are 1 kg per day lower than the current scenario, and 44 kg per cow per year less if production levels are 1 kg per day higher than the current scenario. Thus, assuming that the impact of DP length on milk yield per day is absolute, the impact of shortening or omitting the DP on milk yield per year will be lower on herds with a higher average production level.

The economic impact of shortening or omitting the DP at the herd level was assessed with revenues from sold milk, meat from culled cows and surplus calves, and costs associated with buying or producing feed, and rearing youngstock. Compared with a DP of 56 days, a DP of 28 days reduced partial cash flows by €1,249 per herd per year, and a DP of 0 days reduced partial cash flows by €1,632 per herd per year in the first 5 years of the strategy. This seems to be a limited burden compared with the average Dutch dairy farmer’s family labour income from 2008 to 2016 of €42,322 [55]. Santschi et al. [21] previously reported an increase in net annual income when a DP of 35 days was applied (for one lactation) instead of a DP of 60 days, resulting from an increase of 569 kg in annual milk production per cow. Depending on whether the quota or the number of cows was kept constant, this resulted in an increase of net annual income of $41 (Can$) or $245 per cow. In the current study, annual milk production per cow was 379 kg lower for a DP of 28 days than for a DP of 56 days. Lowering reductions in milk yield after a DP of 28 of 0 days by 1 kg or 2 kg milk per day, however, increased partial cash flows compared with a DP of 56 days by €33 and €101 per cow in year 2.

Partial cash flows were sensitive to assumptions about CI and milk production levels. If a DP of 0 days did not result in a shortened CI, this further reduced partial cash flows by on average €4,498 per herd per year from year 2 onwards. A change in reductions in milk production of 1 kg per day in lactation changed the average partial cash flows by about €6,000 to €7,000 per herd per year. General culling rate had a small impact on partial cash flows. This result depends on the milk price, meat price and rearing costs. In case a mature cow (assumed weight of 650 kg) is culled and slaughtered, for example, revenues for meat are €905, which is only €64 below the assumed rearing costs of the replacement heifer. In reality, costs of culling are likely higher due to costs of diseases prior to culling.

Effects of DP length on disease incidence are not clear yet from experimental and observational studies [16], and related veterinary costs were therefore not included in the model. Assuming that health and fertility will improve in case of short and no DP, as a consequence of the improved energy balance [5,6], this is the most conservative scenario. Partly, the effect of diseases on milk production was implicitly included in the current model, because milk production was based on actual milk records. Disease costs related to veterinary services or discarded milk, however, were not included. Köpf et al. [17] reported €103 lower costs per lactation for treatment of diseases after no DP or spontaneous dry-off than after a DP of 56 days in German Simmental cows. Mostert et al. [53] estimated the costs of subclinical ketosis–with an incidence of 25% in the first 30 days after calving–to be €130 per case per year, of which 33% resulted from treatment and discarded milk. If shortening and omitting the DP not only improve metabolic status, but also reduce the incidence of (subclinical) metabolic disorders, such reductions in costs might easily offset the reductions in partial cash flow due to a short or no DP. In addition, costs related to reproductive treatments and fertility culling may be reduced when the DP is shortened or omitted. Multiple studies report shortened CI, that could be explained by an earlier onset of ovulation and normal overian cyclicity after calving [13–15,56]. Gumen et al. [14] also found that the number of services per conception was lower for cows with no DP (1.75) than for cows with a standard DP (3.00), with cows with a short DP being intermediate (2.44). Assuming €20 per service [18], shortening and omitting the DP could reduce reproductive costs in a herd of 100 cows by more than €1,000 and €2,000 per year, respectively.

The impact of DP length on GHG emissions related to milk production was assessed by calculating GHG emissions per t FPCM. In the current model, GHG emissions per t FPCM on average increased by 8 kg CO_2_ equivalents in case of a DP of 28 days and 5 kg CO_2_ equivalents in case of a DP of 0 days compared with a DP of 56 days. This increase seems small compared with the impact of culling: a reduction in culling rate of 15% reduced average GHG emissions by 56 to 70 kg CO_2_ equivalents per t FPCM between years and DP length strategies. This is comparable to results reported by Van Middelaar et al. [29], who estimated that an increase in lifespan of 270 days–which reduced culling by about 5%–reduced GHG emissions by 23 kg CO_2_ equivalents per t FPCM. In case a change in DP length from 56 days to 0 or 28 days would reduce culling rate by 5%, GHG emissions of milk production would be lower for a DP of 28 days and lowest for a DP of 0 days, compared with DP of 56 days. Opposed to the economic impact, where replacement of a full-grown cow with a heifer costs merely €64, GHG emissions related to rearing a heifer (4,905 kg CO_2_) are much larger than the amount of GHG of meat production that are substituted by slaughtering the cow (2,795 kg CO_2_). If the improved metabolic health reduces the probability of culling in case of a short or no DP, and consequently lengthens the lifespan of dairy cows, the dilution of GHG emissions related to rearing would offset the negative impact on GHG emissions.

In the current study, the impact of DP length on disease incidence and treatment, and its effect on GHG emissions of milk production, was not included. The treatment of diseases is likely to increase GHG emissions per unit milk through discarded milk and removal of cows [53]. Discarded milk due to the use of antibiotics was shown to contribute 30% to the impact of subclinical ketosis on GHG emissions [53]. With a lower milk production per day, and perhaps fewer treatments per lactation in case of reduced disease incidence, less milk may be discarded in case of shortening or omitting the DP, which could reduce GHG emissions of milk produced.

The model used one average ration for all dairy cows, instead of a DP ration and a lactation ration, because the best estimate of the average Dutch ration is only available for all dairy cows together [36]. The ration modification for cows with a DP of 28 or 0 days was based on the assumption that the reduction in energy requirement per day could be matched by a reduction in concentrate of 0.3 kg per cow per day. This amount is comparable to reducing the amount of concentrate by 1.8 kg per cow per day in early lactation and providing an additional 1.0 kg per cow per day in the 8 weeks before calving. Reducing the concentrate availability for cows after a DP of 0 days according to this scheme did not cause a further reduction in milk production, compared with cows with a DP of 0 days that were fed a standard concentrate level [38]. The reduced concentrate ration reduced feed costs at the herd level (- €132 per herd per year) and GHG emissions of milk production (-4 kg CO_2_ equivalents per t FPCM).

The model is a simplification of reality in which we aimed to incorporate and assess scientifically demonstrated effects of shortening or omitting the DP at herd level. Stochastic elements were related to individual lactation potential, CI, and the probability and the moment of culling. Lactation curves and CI were derived from data of commercial dairy farms.

Correlations among stochastic elements were not modelled. A relation between milk yield and culling would be difficult to quantify and requires many assumptions: physiologically, milk yield is related to metabolic status [57] and impaired metabolic status is related to increased culling in early lactation [58]; whereas due to management decisions, the probability of culling increases with lower productivity [59,60]. The probability of culling for fertility reasons was linked to DP length based on the commercial data, and the impact of a change in culling probability was assessed in the sensitivity analysis.

Further variation could be modelled through individual lactation curves, or through a variable delay in replacement of culled cows. Although the simplifications in the current model reduce variation between daily productions of individual cows and cow spaces, they are not expected to change the comparison of yearly productions between herds with different DP lengths.

The evaluation of partial cash flows and GHG emissions was performed with fixed numbers, based on average costs and revenues, Dutch national inventory reports on GHG emissions and IPCC emission factors. These parameter values, however, are variable and uncertain. The current study gives an indication of how shortening or omitting the DP will affect partial cash flows at the herd level and GHG emissions per unit of milk. Higher GHG emissions per unit feed or higher emission factors will increase GHG emissions per unit of milk produced for all DP lengths, but are unlikely to affect the overall comparison between DP lengths. For individual farms, however, farm-specific values should be used to come to a farm-specific conclusion.

Extensions to the model could be the incorporation of specific diseases and treatment of diseases to gain insight in the potential effect of DP length on discarded milk and the consequences for revenues and GHG emissions. Moreover, the model could be adapted to assess the impact of shortening or omitting the DP in seasonal calving systems, where fertility is of greater priority.

The current model results suggest that shortening or omitting the DP negatively affected partial cash flows and GHG emissions; however, considering the small effect size and the potential for enhancing cow welfare [4,9], these negative effects seem justifiable. Variation in effects of DP length on milk production and fertility between farms and overall production level may change these conclusions for individual farms [13,61]. Besides an improvement in cow health, there could be other motivations to shorten or omit the DP. Dutch farmers appreciated the easier management with one ration for all cows, no regrouping, and no drying-off procedure when the DP was omitted [54]. The perceived easier management is not necessarily reflected in reduced labour, because more cows have to be milked.

## Conclusions

Shortening the dry period reduced milk production of the herd by 3.1% from the second year onwards, relative to a conventional dry period. Omitting the dry period reduced milk production of the herd by 3.5% from the third year onwards, after a dip in milk production of 6.9% in the second year. On average over 5 years, short and no dry periods reduced partial cash flows by €1,249 and €1,632 per herd per year, and increased greenhouse gas emissions per kg of milk by 0.8% and 0.5%, respectively, which might be offset by lower disease costs and reduced culling. Considering the potential for enhancing cow welfare, these negative impacts of a short or no dry period seem justifiable.

## Supporting information

S1 Table **Standard errors and P-values for fitted parameters a, b, and c of the lactation curves**.
(DOCX)

S2 Table Emission factors for N_2_O and CH_4_ emissions from manure, on pasture and in stables. (DOCX)
