# Correction: Effects of dry period length on production, cash flows and greenhouse gas emissions of the dairy herd: A dynamic stochastic simulation model

**DOI:** 10.1371/journal.pone.0197872

**Published:** 2018-05-25

**Authors:** The PLOS ONE Staff

There is an error in reference 11. The correct reference is: Kok A, van Knegsel ATM, van Middelaar CE, Engel B, Hogeveen H, Kemp B, et al. Effect of dry period length on milk yield over multiple lactations. J Dairy Sci. 2017;100: 739–49. doi: 10.3168/jds.2016-10963

There is an error in reference 12. The correct reference is: Chen J, Kok A, Remmelink GJ, Gross JJ, Bruckmaier RM, Kemp B, et al. Effects of dry period length and dietary energy source on lactation curve characteristics over 2 consecutive lactations. J Dairy Sci. 2016;99: 9287–99. doi: 10.3168/jds.2016-11253

There is an error in reference 23. The correct reference is: Steffen W, Richardson K, Rockstrom J, Cornell SE, Fetzer I, Bennett EM, et al. Planetary boundaries: Guiding human development on a changing planet. Science. 2015;347: 1259855. doi: 10.1126/science.1259855

There is an error in reference 29. The correct reference is: van Middelaar CE, Berentsen PBM, Dijkstra J, van Arendonk JAM, de Boer IJM. Methods to determine the relative value of genetic traits in dairy cows to reduce greenhouse gas emissions along the chain. J Dairy Sci. 2014;97: 5191–205. doi: 10.3168/jds.2013-7413

There is an error in reference 53. The correct reference is: Mostert PF, Middelaar CE van, Bokkers EAM, Boer IJM de. The impact of subclinical ketosis in dairy cows on greenhouse gas emissions of milk production. J Clean Prod. 2017;171: 773–82. doi: 10.1016/j.jclepro.2017.10.019

The publisher apologizes for the errors.
