# Supplementary material for: Effects of dry period length on production, cash flows and greenhouse gas emissions of the dairy herd: A dynamic stochastic simulation model
Source: PLoS One. 2017 Oct 27;12(10):e0187101. doi: 10.1371/journal.pone.0187101 (PMC5659778; doi:10.1371/journal.pone.0187101)
Supplement: S1 Table — Standard errors and P-values for fitted parameters a, b, and c of the lactation curves. (DOCX) [file pone.0187101.s001.docx]

**S1 Table. Standard errors and P-values for fitted parameters a, b, and c of the lactation curves.**

| Parameter | Component | Estimate | SE | df | P |
| --- | --- | --- | --- | --- | --- |
| a | Intercept | 20.9226 | 0.3643 | 6654 | <.0001 |
|  | Par 1 | -8.9916 | 0.2672 | 7922 | <.0001 |
|  | Par 2 – standard DP | -0.00119 | 0.2797 | 7843 | 0.9966 |
|  | Par 2 – short DP | -2.9724 | 0.3071 | 7654 | <.0001 |
|  | Par 2 – no DP | -6.7875 | 0.3195 | 7444 | <.0001 |
|  | Par >2 – standard DP | 3.5600 | 0.2393 | 5856 | <.0001 |
|  | Par >2 – short DP | 0.7425 | 0.2807 | 5957 | 0.0082 |
|  | Par >2 – no DP 1^st^ time | -2.5319 | 0.3433 | 5840 | <.0001 |
|  | Par >2 – no DP 2^nd^ time | 0 | . | . | . |
|  | Covariate^a^ | 0.002543 | 0.000037 | 5672 | <.0001 |
| b | Intercept | -0.08350 | 0.000540 | 6840 | <.0001 |
|  | Par 1 | 0.03878 | 0.000755 | 5688 | <.0001 |
|  | Par 2 | 0.01274 | 0.000756 | 5697 | <.0001 |
|  | Par >2 | 0 | . | . | . |
| c |  | -16.0916 | 0.1971 | 3550 | <.0001 |

^a^First parity 305 yield in kg fat-and-protein-corrected milk.
