# Supplementary material for: Effects of dry period length on production, cash flows and greenhouse gas emissions of the dairy herd: A dynamic stochastic simulation model
Source: PLoS One. 2017 Oct 27;12(10):e0187101. doi: 10.1371/journal.pone.0187101 (PMC5659778; doi:10.1371/journal.pone.0187101)
Supplement: S2 Table — (DOCX) [file pone.0187101.s002.docx]

**S2 Table. Emission factors for N_2_O and CH_4_ emissions from manure, on pasture and in stables.**

| Emission factors | Pasture | Stable |
| --- | --- | --- |
| N_2_O-N direct | 0.0330 kg/ kg N^a^ | 0.0015 kg/ kg TAN^e,1^ |
| NH_3_-N | 0.0530 kg/ kg TAN^a,1,2^ | 0.1000 kg/ kg TAN^e^ |
| NO_x_-N | 0.0120 kg/ kg N^a^ | 0.0015 kg/ kg TAN^e^ |
| NO_3_-N leaching | 0.1200 kg/ kg N^b^ |  |
| CH_4_ | 0.1100 kg/ m^3^ manure^c^ | 0.7460 kg/ t manure^c^ |
| N_2_O-N via NH_3_ | 0.0100 kg/ kg NH_3_-N^d^ | 0.0100 kg/ kg NH_3_-N^d^ |
| N_2_O-N via NO_x_ | 0.0100 kg/ kg NO_x_-N^d^ | 0.0100 kg/ kg NO_x_-N^d^ |
| N_2_O-N via NO_3_^-^ | 0.0075 kg/ kg NO_3_^-^-N^d^ |  |

References: a: [47]; b: [46]; c [44]; d [51]; e: [45]

^1^TAN = Total Ammoniacal Nitrogen

^2^calculated value: 1.98 × 10^-5^ × (N-content ration)^3.664^; N-content of the ration in summer (i.e. when on pasture) is 30.31 g/ kg DM
